# Comparison of three doses of estradiol benzoate for synchronization of follicular wave emergence in suckled *Bos indicus* beef cows

**DOI:** 10.1590/1984-3143-AR2021-0016

**Published:** 2021-08-25

**Authors:** Amanda Guimarães Silva, Leonardo Marin Ferreira Pinto, Nadark de Amorim Silva, Ana Clara Degan Mattos, Pablo Henrique Ambrósio, Keila Maria Roncato Duarte, Rafael Herrera Alvarez, Guilherme Pugliesi

**Affiliations:** 1 Departamento de Reprodução Animal, Faculdade de Medicina Veterinária e Zootecnia, Universidade de São Paulo, Pirassununga, SP, Brasil; 2 Faculdade de Ciências Agrárias e Veterinárias, Universidade Estadual Paulista “Júlio de Mesquita Filho”, Jaboticabal, SP, Brasil; 3 Unidade de Pesquisa e Desenvolvimento Tietê, Agência Paulista de Tecnologia dos Agronegócios, Tietê, SP, Brasil

**Keywords:** Bos indicus, cattle, estradiol benzoate, follicular dynamics, multiparous, primiparous

## Abstract

We aimed to compare the effect of three estradiol benzoate (EB) doses on follicular wave emergence (FWE) and dominant follicle growth of suckled Nelore cows submitted to TAI (D0). On a random day of estrous cycle (D−10), multiparous (MULT; n=36) and primiparous (PRIM; n=20) suckled Nelore cows received an intravaginal progesterone (P4) device and were assigned in three groups. Cows in the EB-1 (n=20), EB-1.5 (n=15) or EB-2 (n=21) groups received, respectively, an im treatment with 1, 1.5 or 2 mg EB. A subgroup (n=10-13 cows/group) were subject to daily ovarian evaluations from D−10 to D0. On D−2, P4 devices were removed, and all cows received the same treatment: 1 mg estradiol cypionate, 0.53 mg sodium cloprostenol, and 300 IU eCG. Statistical analyses were performed considering only the main effects of treatment group and parity order. The proportion of cows with a synchronized FWE and the moment of the FWE did not differ (*p*>0.05) among the treatment groups (overall: 80% [28/35] and 4.1 ± 0.4 days); however, the FWE occurred earlier (*p*=0.007) in MULT (3.8 ± 0.2 days) than PRIM (5.1 ± 0.4) cows. The proportion of animals detected in estrus was greater (86% [31/36] *vs.* 70% [14/20]; *p*=0.02) and the dominant follicle was larger on D−2 (9.7 ± 0.3 mm *vs.* 7.8 ± 0.7 mm; *p*=0.006) and D0 (11.9 ± 0.4 mm *vs.* 10 ± 0.5 mm; *p*=0.008) in MULT than PRIM cows. In conclusion, the three EB doses presented similar efficiency to synchronize the FWE in suckled Nelore cows. Moreover, a delayed FWE and smaller dominant follicle is observed in PRIM cows, contributing to the reduced reproductive performance in this parity category when using similar TAI protocols of MULT cows.

## Introduction

Timed artificial insemination (TAI) programs have been applied routinely on the last years in dairy and beef cattle herds, enabling the spread of artificial insemination (Bisinotto and Santos, 2011). The estradiol (E2)/progesterone (P4)-based protocols have been the most used for *Bos indicus* or *crossbreed Bos indicus* females in the tropical and sub-tropical regions, as this association has resulted in better pregnancy outcomes than the GnRH-based protocols (Baruselli et al., 2012). The association of E2 and P4 at the beginning of the protocol for TAI promotes atresia of the ovarian follicles and a new follicular wave emergence (FWE) (Bó et al., 2003).

The E2 benzoate (EB) is the most commonly used E2 ester at the beginning of the E2/P4-based protocols (Bó et al., 1995; Burke et al., 2001, 2003; Martínez et al., 2005; Sá Filho et al., 2006, 2011). The EB treatment induces in a dose-dependent manner the atresia of FSH-dependent follicles and a new FWE between 2 to 6 days post-treatment (Burke et al., 2003; Bastos et al., 2011). Although the conventional EB dose used at the beginning of the TAI protocol in suckled beef cows is 2 mg, a lower EB dose (1 mg) has been indicated in beef heifers, as increased circulating E2 concentrations are observed in animals with lesser body weight and liver clearance (Bó et al., 2002; Martínez et al., 2005). A recent study (Pessoa et al., 2015) reported a more synchronized FWE in *Bos indicus* and *Bos taurus* suckled beef cows receiving 2 mg than 1 mg EB for resynchronization of ovulation 22 days after TAI. However, the use of 2 mg EB compared to 1mg EB in resynchronization protocols starting 14 days post-TAI in beef suckled cows results in greater pregnancy loss of the first TAI (Silva et al., 2020). Therefore, an intermediate EB dose could be more adequate to increase the FWE synchrony for a second TAI and preferred to avoid the risk of pregnancy loss, but there is no study evaluating whether the use of an EB dose between 1 and 2 mg can efficiently synchronize FWE. Also, for a better optimization of the TAI protocols, the differential effects of the EB/P4-based protocol on synchronization of FWE between multiparous and primiparous suckled cows have to be determined. In this regard, primiparous beef cows have a lower reproductive performance and a greater nutritional demands compared to multiparous cows (Sá Filho et al., 2009, 2010, 2013; Sales et al., 2016).

In the present study, we hypothesized that in suckled *Bos indicus* beef cows: 1 mg EB would not be efficient to synchronize a new FWE, but 1.5 mg EB effectively synchronize the FWE as 2 mg. Therefore, we aimed to compare the ovarian follicular dynamics in primiparous and multiparous *Bos indicus* suckled beef cows receiving three different EB doses (1, 1.5 and 2 mg) for synchronization of FWE in a TAI protocol.

## Materials and methods

### Animals and experimental design

**Figure 1:**
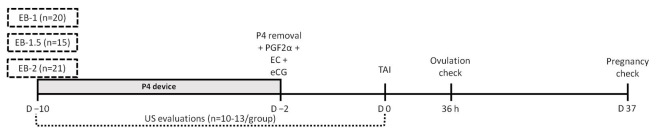
Schematic diagram of the experimental design. Suckled Nelore cows were submitted to a E2/P4 based TAI protocol and on D−10 were alocated in three groups: EB-1 (1 mg EB), EB-1.5 (1.5 mg EB) or EB-2 (2 mg EB) and all females reiceved a eight days-used P4 device. A subgroup (n=10-13/group) were submitted to daily (D−10 to D0) ultrassonography evaluations (B-mode). On D−2, the devices were removed and all cows received 1 mg E2 cypionate (EC), 0.53 mg sodium cloprostenol (PGF2α), and 300 IU equine chorionic gonadotrophin (eCG). The TAI was performed 48 hours after the treatment for induction of ovulation and the ovulation was checked 36 hours after TAI. The pregnancy diagnosis was performed through ultrasound B-mode on D37.

The experiment was approved by the ethics committee of the School of Veterinary Medicine and Animal Science of the University of São Paulo (protocol 3851080519) and was carried out at the São Paulo Agribusiness Technology Agency (Tietê, SP, Brazil). Thirty-six multiparous and 20 primiparous suckled *Bos indicus* cows (Nelore), 160 ± 8.2 days postpartum and with an average body condition score (BCS) 2.99 ± 0.39 (1 to 5 scale; 1 [emaciated] and 5 [obese]) were used. On a random day of estrous cycle animals were submitted to an ovulation synchronization protocol (Figure 1). On day −10 (D−10), cows received an intravaginal P4-releasing device (Sincrogest®, Ourofino Saúde Animal, Brazil) and were randomly assigned to either i.m. EB treatment: 1 mg EB (Sincrodiol®, Ourofino; EB-1 [n=20, 12 multiparous and 8 primiparous]), 1.5 mg EB (EB-1.5 [n=15, 9 multiparous and 6 primiparous]) or 2 mg EB (EB-2 [n=21, 15 multiparous and 6 primiparous]). On D−2, the devices were withdrawn, and regardless of treatment group, all cows received: 1 mg E2 cypionate (1mL; i.m., SincroCP®, Ourofino), 0.53 mg sodium cloprostenol (2 mL; i.m., Cioprostinn®, Boehringer-Ingelheim), and 300 IU equine chorionic gonadotrophin (1.5 mL; i.m., eCG; SincroeCG®, Ourofino). Cows were painted with chalk marker halfway between the hip and tail head to determine the occurrence of estrus between D−2 and D0. On D0, TAI was performed by a single technician with thawed semen from two sires.

A subgroup of multiparous [EB-1, n=8; EB-1.5, n=9; and EB-2, n=8] and primiparous [EB-1, n=4; EB-1.5, n=4; and EB-2, n=2] cows was subjected to daily ovarian evaluations by ultrasonography from D−10 to D0. Scanning was performed by a single operator using an ultrasound instrument (DP50-VET, Mindray, China) in B-mode with a linear multifrequency probe. Multiparous with a detected new FWE occurring from 3 to 5 days and primiparous with a detected new FWE occurring from 4 to 6 days after the treatment were considered to have a synchronized follicle wave emergence and the proportion of cows with a synchronized wave was calculated. The day of the new FWE was defined by retrospective evaluation, when the dominant follicle (DF) first appeared between 4 and 5 mm on the ultrasound image (Ginther et al., 1989). The follicular diameter was calculated by the mean of the maximum length and width using the caliper function. For estrous evaluations, they were scored from 0 to 3, based on the color change between D−2 and D0, in which 0 = unchanged, 1 < 50% of color removed, 2 > 50% of color removed and 3 = 100% of color removed. The occurrence of estrus was defined when the paint was classified as 2 or 3. Ovulation was confirmed by disappearance of DF at the ultrasonographic exam 36 h after TAI. Pregnancy diagnosis was done on D37 by transrectal ultrasonography to detect the presence of a viable embryo with heartbeat.

### Statistical analyses

Statistical analyses were performed using SAS software (version 9.2, SAS Institute Inc., USA). The continuous dependent variables (day of FWE, follicular growth rate and DF size) were evaluated for the normality of residues by the Shapiro-Wilk test and homogeneity of variances by Levene’s test. Data were analyzed using ANOVA with the MIXED procedure considering only the mains effects of treatment group (EB doses) and parity order, as the reduced number of primiparous cows in each treatment group limited the evaluation of a possible interaction between treatment and parity. The dispersion of the day of FWE was analyzed by Bartlett’s test. The frequency of a new FWE that occurred from 3 to 5 days (multiparous) or 4 to 6 days (primiparous) post-treatment and the estrous, ovulation and pregnancy rates were compared by Fisher’s exact test using FREQ procedure of SAS. Results are presented as mean ± SEM and proportion. Probabilities of *p* ≤ 0.05 indicate significant difference.

## Results

**Figure 2:**
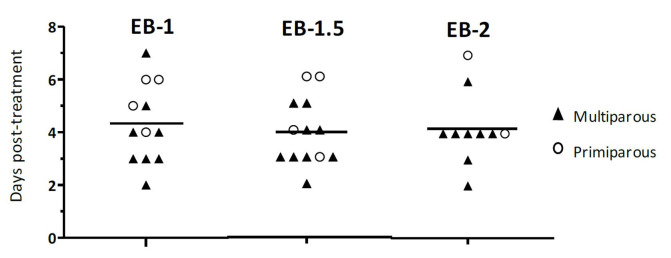
Individual values to day of follicular wave emergence in multiparous (▲) and primiparous (○) cows. The horizontal bars represent the aritmetric mean for each treatment. The dispersion of the detected day of FWE did not differ among the treatment groups when analyzed by Bartlett’s test (p > 0.05).

A new FWE was detected in all animal’s subject to daily evaluations (n=35 animals). There was no difference (*p* > 0.05) on the mean day of FWE among the treatment groups (Table 1); however, a parity effect (*p* = 0.007) indicated a delayed FWE in primiparous than multiparous cows. When the dispersion of the detected day of FWE was analyzed by the Bartlett’s test, no difference (*p* > 0.05) was detected among the treatment groups or parity categories (Figure 2). Also, the proportion of cows with a synchronized FWE (multiparous, from 3 to 5 days post-treatment; and primiparous, from 4 to 6 days post-treatment) did not differ (*p* > 0.05) among the treatment groups (83% [10/12], 85% [11/13] and 70% [7/10], for EB-1, EB-1.5 and EB-2 groups, respectively).

**Table 1:** Follicular wave emergence (FWE), follicle growth rate, dominant follicle diameter at TAI of cows (multiparous and primiparous) treated with 1, 1.5, or 2 mg at the beginning of the TAI protocol. All values are expressed as mean ± SEM or proportions.

	**EB groups†**			Parity category*		***P* value**	
	**EB-1 (n=20)**	**EB-1.5 (n=15)**	**EB-2 (n=21)**	**PRIM (n=20)**	**MULT (n=36)**	**G**	**C**
Follicular wave emergence, days †*	4.3 ± 0.4	3.9 ± 0.3	4.2 ± 0.4	5.1 ± 0.4	3.8 ± 0.2	NS	0.007
Follicle growth rate from FWE to TAI, mm/day†*	1.24 ± 0.4	1.19 ± 0.08	1.10 ± 0.07	1.06 ± 0.05	1.26 ± 0.24	NS	NS
Diameter of largest follicle at D−2, mm	9.1 ± 0.5	9.5 ± 0.5	8.6 ± 0.7	7.8 ± 0.7	9.7 ± 0.3	NS	0.006
Diameter of largest follicle at TAI, mm	11 ± 0.6	11.8 ± 0.6	11.1 ± 0.6	10 ± 0.5	11.9 ± 0.4	NS	0.008
Cows detected in estrus at TAI, %	85 [17/20]	67 [10/15]	86 [18/21]	70 [14/20]	86 [31/36]	NS	0.02
Ovulation rate, %	75 [15/20]	67 [10/15]	57 [12/21]	55 [11/20]	72 [26/36]	NS	NS
Pregnancy rate, %	50 [10/20]	40 [6/15]	33 [7/21]	40 [8/20]	42 [15/36]	NS	NS

† Number of cows evaluated by daily ultrasonographic evaluations in each group, EB-1 (n=12), EB-1.5 (n=13) and EB-2 (n=10); *Number of cows evaluated by daily ultrasonographic evaluations in each parity order category, PRIM (n=10) and MULT (n=25); FWE= follicular wave emergence; TAI= timed artificial insemination; PRIM = primiparous; MULT = multiparous; G= Group effect; C: category effect; NS= Nonsignificant (P>0. 05).

The DF diameter (Table 1) was smaller in primiparous cows on D−2 (*p* = 0.006) and at TAI (*p* = 0.008) but did not differ (*p* > 0.05) among the EB treatment groups. The proportion of cows detected in estrus at TAI did not differ (p > 0.05) among the treatment groups (Table 1), but a category effect (*p* = 0.02) reflected a greater proportion of multiparous cows detected in estrus than primiparous cows. No difference (*p* > 0.05) was observed for the follicle growth rate (mm/day) from FWE to TAI among the EB treatment groups nor between primiparous and multiparous cows (Table 1). Also, no effect (*p* > 0.05) of treatment group or parity was observed on ovulation and pregnancy rates (Table 1).

## Discussion

In the present study, three EB doses (1, 1.5 and 2 mg) were used simultaneously for the first time to compare its effects on FWE and DF characteristics in suckled Nelore cows, aiming to define which EB dose is better to effectively synchronize FWE in suckled Nelore cows. A control group (without EB treatment) was not included as the objective was compare the doses already used in TAI protocols and where the insertion of the P4 device without E2 administration is known to lead to follicular persistence (Kinder et al., 1996). Also, a limitation of the herein study is that an interaction between EB dose and parity order was not evaluated due to the limited number of primiparous cows receiving each EB dose. Our expectation that 1 mg EB would not be efficient to synchronize a new FWE in suckled beef cows was not supported. The FWE was not affected by the EB dose used at the beginning of the protocol and 83% [10/12] of cows that received 1mg EB had a synchronized FWE (FWE occurring in a range of three days). A similar efficacy of 1 mg EB compared to 2 mg has been previously reported in suckled *Bos taurus* and *Bos indicus* beef cows and *Bos taurus* dairy cows, both non-lactating (Burke et al., 2003; Bastos et al., 2011). But, when these both doses were in *Bos indicus* suckled cows for resynchronization on day 22 after TAI (Pessoa et al., 2015), the FWE occurred more dispersed in cows treated with 1 mg EB. This divergence between the previous and present results may be related to several factors affecting circulating E2 concentrations at the time of EB treatment, as the size of DF and the liver clearance of steroid hormones. According to Sartori et al. (2016), *Bos indicus* cows have a slower metabolism of steroid hormones, compared to *Bos taurus* cows. In addition, increased clearance of steroid hormones is directly correlated with high dry matter intake and lactation (Sangsritavong et al., 2002). Thus, the 1 mg EB dose at the beginning of the protocol may be appropriate to synchronize the FWE in cows kept on pastures without feed supplementation or in late postpartum period (reduced milk yield), as well as in the present study.

Although the comparison of intermediate EB doses as 1.5 mg with other doses has not been reported for *Bos indicus* females, a study with suckled Hereford and Hereford x Charolais crossbreed (Pfeifer et al., 2018) using 1.5 mg EB associated to a 0.78 g P4 device reported that only 52.4% [11/21] of suckled beef cows had follicular atresia and a new FWE. The results obtained with the present study supported our hypothesis that 1.5 mg EB effectively synchronize the FWE in *Bos indicus* beef cows, regardless the parity order. That is, the dose of 1.5 mg induced a new FWE between 3 to 6 days after the EB treatment in 75% of primiparous and 88% of multiparous cows. Also, similar DF size, proportion of cows detected in estrus and ovulation and pregnancy rates were observed in cows treated with 1.5 or 2 mg EB.

Regarding the parity effect on follicular dynamics, the present study indicated one day delay in FWE and a reduced DF size at P4 device removal and at TAI in primiparous than multiparous Nelore cows. The delay in FWE may be resulting from a longer FSH suppression caused by increased circulating E2 in primiparous than multiparous, as first parous cows at calving are about 85% of adult weight (Diskin and Kenny, 2014). Consequently, primiparous cows are lighter after partum (Ungerfeld et al., 2011), have greater weight loss during early postpartum, and present 7% less body weight than the multiparous (second calving order) at the weaning period (Vieira et al., 2005).

The reduced DF size in primiparous cows could be a direct consequence of the delayed FWE, resulting in a short period for DF growth between follicle deviation to TAI. Still, a reduced gonadotropin support for final DF growth is associated as the primary cause for a smaller DF in primiparous cows, as a lower circulating LH pulsatility and IGF-I concentration is reported in primiparous than multiparous cows (Meikle et al., 2004). Also, primiparous cows have a greater energy demand than multiparous, as they need energy for lactation and body growing (NRC, 2016). Consequently, a more severe anestrous condition and lower reproductive performance during early postpartum is reported in primiparous than multiparous *Bos indicus* cows subjected to TAI protocols (Sá Filho et al., 2009, 2013; Sales et al., 2016). The delayed FWE and reduced gonadotropin support for follicle grow might be associated for leading to a smaller DF at TAI in primiparous cows, which has a positive and linear relationship with pregnancy rates in TAI programs of beef cattle (Sá Filho et al., 2011; Pugliesi et al., 2016; Nishimura et al., 2018). Therefore, the hormonal protocols for TAI in primiparous suckled cows would be modified to allow an extended period or greater gonadotropin support for follicle grow during the LH-dependent phase aiming to improve reproductive performance. Thus, the reduction in 25 to 50% of the conventional EB dose (2mg) for synchronization of FWE at beginning of the E2/P4-based protocol could be an interesting alternative with high efficiency in induction of a new FWE for situations with lighter cows or for super-early resynchronization protocols, where doses greater than 1 mg EB may negatively affect the previous pregnancy (Silva et al., 2020). Though, because of the limited number of animals used in the present study, further studies are needed to evaluate the effect of reduced EB doses on the pregnancy rate in suckled beef cows.

In conclusion, the EB doses varying from 1 to 2 mg proved to be efficient to synchronize the emergence of a new follicular wave in suckled Nelore cows. The delayed FWE in primiparous cows is a factor contributing to the reduced DF diameter at TAI compared to multiparous cows, indicating that the TAI protocols in first parous cows would be modified to allow an extended time for gonadotropin support from FWE to TAI.

## References

[B001] Baruselli P, Sales JNS, Sala RV, Vieira LM, Sá MF (2012). History, evolution and perspect ives of timed artificial in semination programs in Brazil. Anim Reprod.

[B002] Bastos MR, Surjus RS, Prata AB, Meschiatti MAP, Borsato M, Mourão GB, Pedroso AM, Pires AV, Sartori R (2011). Efeito da dose de benzoato de estradiol em associação à progesterona na sincronização da emergência da onda folicular em vacas Bos indicus e Bos taurus..

[B003] Bisinotto RS, Santos JEP (2011). The use of endocrine treatments to improve pregnancy rates in cattle. Reprod Fertil Dev.

[B004] Bó GA, Adams GP, Caccia M, Martinez M, Pierson RA, Mapletoft RJ (1995). Ovarian follicular wave emergence after treatment with progestogen and estradiol in cattle. Anim Reprod Sci.

[B005] Bó GA, Baruselli PS, Martínez MF (2003). Pattern and manipulation of follicular development in Bos indicus cattle. Anim Reprod Sci.

[B006] Bó GA, Baruselli PS, Moreno D, Cutaia L, Caccia M, Tríbulo R, Tríbulo H, Mapletoft RJ (2002). The control of follicular wave development for self-appointed embryo transfer programs in cattle. Theriogenology.

[B007] Burke CR, Mussard ML, Gasser CL, Grum DE, Day ML (2003). Estradiol benzoate delays new follicular wave emergence in a dose-dependent manner after ablation of the dominant ovarian follicle in cattle. Theriogenology.

[B008] Burke CR, Mussard ML, Grum DE, Day ML (2001). Effects of maturity of the potential ovulatory follicle on induction of oestrus and ovulation in cattle with oestradiol benzoate. Anim Reprod Sci.

[B009] Diskin MG, Kenny DA (2014). Optimising reproductive performance of beef cows and replacement heifers. Animal.

[B010] Ginther OJ, Knopf L, Kastelic JP (1989). Temporal associations among ovarian events in cattle during oestrous cycles with two and three follicular waves. J Reprod Fertil.

[B011] Kinder JE, Kojima FN, Bergfeld EG, Wehrman ME, Fike KE (1996). Progestin and estrogen regulation of pulsatile LH release and development of persistent ovarian follicles in cattle. J Anim Sci.

[B012] Martínez MF, Kastelic JP, Bó GA, Caccia M, Mapletoft RJ (2005). Effects of oestradiol and some of its esters on gonadotrophin release and ovarian follicular dynamics in CIDR-treated beef cattle. Anim Reprod Sci.

[B013] Meikle A, Kulcsar M, Chilliard Y, Febel H, Delavaud C, Cavestany D, Chilibroste P (2004). Effects of parity and body condition at parturition on endocrine and reproductive parameters of the cow. Reproduction.

[B014] NRC (2016). Nutrient requirements of beef cattle.

[B015] Nishimura TK, Martins T, da Silva MI, Lafuente BS, de Garla Maio JR, Binelli M, Pugliesi G, Saran A (2018). Importance of body condition score and ovarian activity on determining the fertility in beef cows supplemented with long-acting progesterone after timed-AI. Anim Reprod Sci.

[B016] Pessoa GA, Martini AP, Chaiben M, Vieira LM, Girotto RW, Pugliesi G, Santin T, Batistella Rubin MI, Baruselli PS, Sá MF (2015). Adjustment of the estradiol benzoate dose in the resynchronization protocol with unknown pregnancy status in suckled beef cows..

[B017] Pfeifer LFM, Mapletoft RJ, Dardawal D, Singh J (2018). Effect of injectable progesterone on follicular development in lactating beef cows treated with estradiol plus a low-concentration progesterone device. Braz J Vet Res Anim Sci.

[B018] Pugliesi G, Santos FB, Lopes E, Nogueira É, Maio JRG, Binelli M (2016). Improved fertility in suckled beef cows ovulating large follicles or supplemented with long-acting progesterone after timed-AI. Theriogenology.

[B019] Sá MF, Reis EL, Ayres H, Gimenes LU, Peres AAC, Carvalho CAB, Carvalho JB, Araujo CASC, Baruselli PS (2006). Effect of oestradiol valerate or benzoate on induction of a new follicular wave emergence in bos indicus cows and heifers treated with norgestomet auricular implant. Reprod Fertil Dev.

[B020] Sá MF, Crespilho AM, Santos JEP, Perry GA, Baruselli PS (2010). Ovarian follicle diameter at timed insemination and estrous response influence likelihood of ovulation and pregnancy after estrous synchronization with progesterone or progestin-based protocols in suckled Bos indicus cows. Anim Reprod Sci.

[B021] Sá MF, Baldrighi JM, Sales JNS, Crepaldi GA, Carvalho JBP, Bó GA, Baruselli PS (2011). Induction of ovarian follicular wave emergence and ovulation in progestin-based timed artificial insemination protocols for *Bos indicus* cattle. Anim Reprod Sci.

[B022] Sá MF, Penteado L, Reis EL, Reis TANPS, Galvão KN, Baruselli PS (2013). Timed artificial insemination early in the breeding season improves the reproductive performance of suckled beef cows. Theriogenology.

[B023] Sá OJ, Meneghetti M, Peres RFG, Lamb GC, Vasconcelos JLM (2009). Fixed-time artificial insemination with estradiol and progesterone for Bos indicus cows II: strategies and factors affecting fertility. Theriogenology.

[B024] Sales JNS, Bottino MP, Silva LACL, Girotto RW, Massoneto JPM, Souza JC, Baruselli PS (2016). Effects of eCG are more pronounced in primiparous than multiparous Bos indicus cows submitted to a timed artificial insemination protocol. Theriogenology.

[B025] Sangsritavong S, Combs DK, Sartori R, Armentano LE, Wiltbank MC (2002). High feed intake increases liver blood flow and metabolism of progesterone and estradiol-17β in dairy cattle. J Dairy Sci.

[B026] Sartori R, Monteiro PLJ, Wiltbank MC (2016). Endocrine and metabolic differences between Bos taurus and Bos indicus cows and implications for reproductive management. Anim Reprod.

[B027] Silva AG, Nishimura TK, Rocha CC, Motta IG, Laurindo A, Ferraz PA, Orlandi RE, Massoneto JPM, Scandiuzzi LAJ, Pugliesi G (2020). Dose ‐ dependent effects of estradiol benzoate for resynchronization of ovulation at 14 days after timed artificial insemination in beef cows. Anim Reprod.

[B028] Ungerfeld R, Hötzel MJ, Scarsi A, Quintans G (2011). Behavioral and physiological changes in early-weaned multiparous and primiparous beef cows. Animal.

[B029] Vieira A, Lobato JFP, Torres RAA, Cezar IM, Correa ES (2005). Fatores determinantes do desempenho reprodutivo de vacas Nelore na região dos Cerrados do Brasil Central. Rev Bras Zootec.

